# Does intraoperative bone density testing correlate with parameters of primary implant stability? A pilot study in minipigs

**DOI:** 10.1002/cre2.224

**Published:** 2019-09-30

**Authors:** Tanja Grobecker‐Karl, Victor Palarie, Sonja Schneider, Matthias Karl

**Affiliations:** ^1^ Department of Prosthodontics Saarland University Homburg Germany; ^2^ Department of Oral and Maxillofacial Surgery and Oral Implantology "A. Gutan" University of Medicine and Pharmacy "N. Testemitanu" Chisinau Moldova

**Keywords:** bone density, dental implantation, osteotomy, torque

## Abstract

**Objectives:**

Bone density, surgical protocol, and implant design are the major determinants of primary stability. The goal of this animal trial was to investigate potential correlations of intraoperative bone density testing with clinical and histologic parameters of primary implant stability.

**Material and methods:**

Following extractions of all mandibular premolars and subsequent healing, four implants each were placed in a total of four minipigs. Bone density was determined by applying intraoperative compressive tests using a device named BoneProbe whereas measurements of implant insertion torque and resonance frequency analysis were used for evaluating implant stability. Bone mineral density (BMD) and bone to implant contact were quantified after harvesting mandibular block sections. Spearman rank correlation tests were performed for evaluating correlations (α = .05).

**Results:**

Due to variation in clinical measurements, only weak correlations could be identified. A positive correlation was found between the parameters bone to implant contact and BMD (Spearman's rho .53; *p =* .05) whereas an inverse correlation was observed between BMD and implant stability (Spearman's rho −.61; *p =* .03). Both BoneProbe measurements in the cortical and trabecular area positively correlated with implant insertion torque (Spearman's rho 0.60; *p =* .02). A slightly stronger correlation was observed between the average of both BoneProbe measurements and implant insertion torque (Spearman's rho.66; *p =* .01).

**Conclusions:**

While establishing exact relationships among parameters of implant stability and the measurement techniques applied would require greater sample size, intraoperative compressive testing of bone might, despite the weak correlations seen here, be a useful tool for predicting primary implant stability.

## 1 INTRODUCTION

Primary stability represents the first step for successful osseointegration (Falco, Berardini, & Trisi, 2018) and appears to be a function of bone density (Karl, Palarie, Nacu, & Krafft, 2013), surgical protocol (Degidi, Daprile, & Piattelli, 2017), and implant design (Menicucci, Pachie, Lorenzetti, Migliaretti, & Carossa, 2012, Coelho et al., 2015, Wilson, Miller, Trushkowsky, & Dard, 2016). A classic measure for primary stability is maximum implant insertion torque, which has been shown to be sensitive with respect to osteotomy size resulting in higher values in undersized recipient sites (Marin et al., 2016). Similarly, resonance frequency analysis of dental implants (Di Stefano, Arosio, Gastaldi, & Gherlone, 2018; Huang et al., 2011) has been frequently applied as an alternative measure. For both variables, contradicting findings with respect to clinical relevance can be found (Falco et al., 2018; Sierra‐Rebolledo, Allais‐Leon, Maurette‐O'Brien, & Gay‐Escoda, 2016), and consequently, insertion energy, defined as the area under the torque‐curve over time during implant insertion, has been advocated (Degidi et al., 2017; Degidi, Daprile, Piattelli, & Iezzi, 2013; Di Stefano et al., 2018) as a dynamic measure. All these measurements have in common that they are applied in a retrospective fashion, that is, the implant has already been installed, and alterations in the surgical protocol are no longer possible.

Although high levels of insertion torque have been recommended for immediate loading protocols due to lower micromotion at the implant bone interface (Trisi, Todisco, Consolo, & Travaglini, 2011), potentially negative effects resulting from the associated bone damage and microfractures leading to implant stability loss at compression sites (Wilson et al., 2016) and bone resorption (Cha et al., 2015) have been discussed. In this context, it has been shown that also trabecular bone may contribute to primary implant stability (Dorogoy, Rittel, Shemtov‐Yona, & Korabi, 2017) whereas cortical bone may resorb if implants cause too much strain during insertion (Eom et al., 2016). It could be shown in a clinical study that overstressing cortical bone by inserting a tapered implant can result in greater likelihood of failure (Menicucci et al., 2012). Similarly, Duyck and coworkers showed that lower levels of insertion torque applied on dental implants led to pronounced bone neoformation (Duyck et al., 2015).

Based on a materials law describing the mechanical properties of both cortical and trabecular bone and following Finite Element Simulations, compressive tests of alveolar bone surrounding an implant osteotomy seemed to be suitable for objectively determining bone density (Winter, Krafft, Steinmann, & Karl, 2011). A device named BoneProbe and consisting of a gradually expandable segmented metal cylinder, which could be inserted into an implant osteotomy, was designed and tested in vitro (Krafft, Winter, Wichmann, & Karl, 2012). In principal, the BoneProbe correlates a certain level of sensor expansion with the force needed to reach this level of expansion. Further studies showed that even small changes in bone density induced, for example, by osteotomes could be detected in vitro (Krafft, Graef, Winter, Wichmann, & Karl, 2013), and BoneProbe measurements correlated well with early implant healing in an extraoral animal model (Karl et al., 2013). The latest version of the BoneProbe was designed as an addition to a surgical motor in the form of a modified contra angle handpiece, where the motor could be used for measuring the torque required for a certain level of expansion. Reasonable reliability of this device could be proven in a human cadaver study (Karl, Buder, Krafft, & Grobecker‐Karl, 2019).

The primary goal of this animal experiment was to obtain preliminary BoneProbe values for an intraoral animal model. As a secondary endpoint, intraoperative compressive bone density testing of native alveolar bone was to be correlated with measurements of implant insertion torque and implant stability as well as with bone mineral density (BMD) and bone to implant contact (BIC) as determined by microradiographs and histomorphometry.

## 2 MATERIAL AND METHODS

### 2.1 General course

Following ethics commission approval (Comitetului de Etica a Cercetarii, State Medical and Pharmaceutical University “Nicolae Testemitanu”, Chisinau, Moldova), a total of four minipigs (mean age: 21.8 months; mean body weight: 46.5 kg) were allocated for this study. The animals were kept as a group in a controlled facility.

General anesthesia was induced and maintained using an intravenous administered combination of Diazepam (Diazepam 10mg—Rotexmedica Injektionslösung, Rotexmedica GmbH Arzneimittelwerk, Trittau, Germany), Ketamin (Ketamin‐hameln 50mg/ml, hameln pharma plus GmbH, Hameln, Germany), and Acepromazine maleate (Castran, Interchemie werken “De Adelaar” B. V., La Waalre, The Netherlands). Heart rate, respiratory rate, O2 saturation, and expiratory CO2 were monitored throughout all surgical procedures (Low Flow Capnograph V900040LF, SurgiVet Inc, Waukesha, WI, USA). Additionally, Ceftriaxon (Ceftriaxon‐ratiopharm, ratiopharm GmbH, Ulm, Germany) and Dexketoprofen (Keral, Menarini International Operations Luxembourg SA, Luxemburg) were used as antibiotic and analgesic, respectively.

Prior to any surgical intervention, local anesthetic was applied (UDS Forte, Sanofi, Frankfurt am Main, Germany) followed by disinfection using chlorhexidine (Chlorhexamed FORTE alkoholfrei 0.2**%,** GlaxoSmithKline Consumer Healthcare, Bühl, Germany). Resorbable 4.0 suture material was used for achieving primary wound closure (Vicryl, Ethicon, Norderstedt, Germany). The animals were fed with soft food and water ad libitum. Sacrificing the animals was carried out by intracardial injection of T61 (0.12‐ml/kg bodyweight; Merck Animal Health, Madison, NJ, USA) as part of the second surgical intervention. Mandibular block sections containing the surgical sites were harvested removing all soft tissue and fixed in neutrally buffered formalin.

### 2.2 Surgical interventions

**Figure 1:**
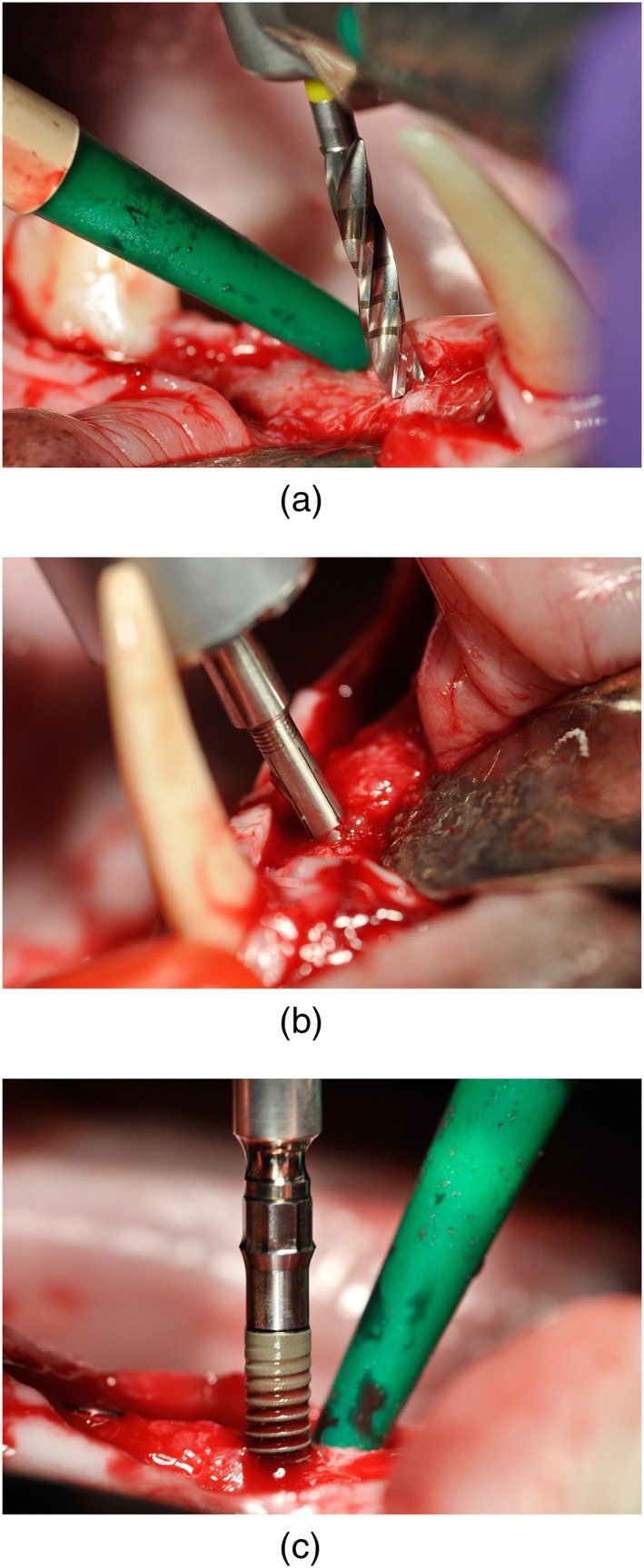
Creation of a 2.8‐mm osteotomy in a healed alveolar ridge (a) was followed by BoneProbe measurements depicted here with the sensing element of the BoneProbe inserted into the drill hole and gradually expanded recording the required torque as a measure of bone density (b). As a final step, a bone level implant was inserted while actively measuring the torque required (c)

The first intervention included the bilateral extraction of all mandibular premolars. Alveolar bone was reduced vertically, bone contours were rounded, and periosteal incisions were made in order to achieve primary closure of the soft tissue. After a healing period of 12 weeks, midcrestal incisions were made and mucoperiosteal flaps spanning the complete edentulous area on both sides of the mandible were carefully reflected. A total of four study sites were subsequently available in each animal. Implant site preparation (Figure 1a) was done using the implant manufacturer's 1.4‐mm‐round burr, 2.2‐mm pilot drill 1, and 2.8‐mm pilot drill 2 (Straumann Bone Level Implant, Straumann GmbH, Freiburg, Germany). Compressive testing of both cortical and trabecular bone (Figure 1b) surrounding the implant site was subsequently performed using the BoneProbe (Karl et al., 2013; Karl et al., 2019; Krafft et al., 2012; Krafft et al., 2013). Following the creation of a 2.8‐mm pilot drill hole, a segmented metal cylinder was inserted and gradually expanded by a surgical motor (iChiropro, Bien‐Air Dental, Biel, Switzerland) whereas the torque needed for expansion was recorded as a measure of bone density. One titanium implant (BoneLevel Implant 3.3 × 8‐mm NC SLActive, Straumann GmbH) was then placed into each study site (Figure 1c) using a surgical motor (iChiropro) for determining maximum implant insertion torque (Sierra‐Rebolledo et al., 2016). Primary implant stability was measured by means of resonance frequency analysis using an Osstell mentor device and implant‐specific SmartPeg abutments (RFA, Osstell AB, Gothenburg, Sweden). All of these measurements were carried out by one single experienced surgeon. As the animals were sacrificed following implant insertion, the wounds were not closed.

### 2.3 Histomorphometric and mircoradiographic analysis

**Figure 2:**
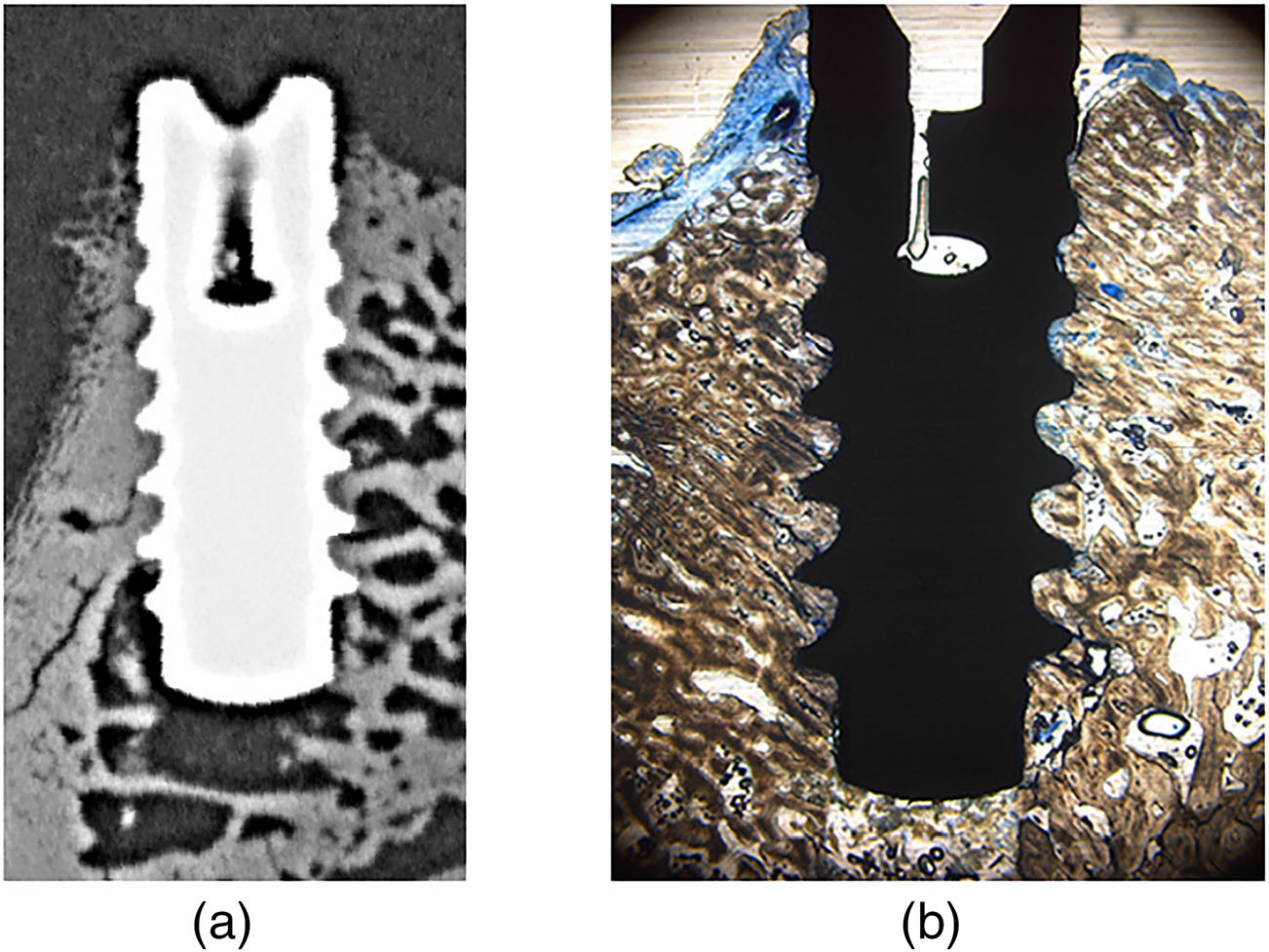
Typical microradiograph (a) and histologic section following toluidine blue staining (b) used for determining bone mineral density and bone to implant contact

All bone specimens were fixed in 10% neutral‐buffered formalin for 48 hr and reduced to rectangular blocks containing the study sites using a diamond band saw (EXAKT 300, EXAKT Advanced Technologies GmbH, Norderstedt, Germany). Subsequently, the specimens were dehydrated in alcohol solutions of increasing concentrations, clarified in xylene and embedded in polymethylmethacrylate (Technovit 9100, Heraeus Kulzer, Hanau, Germany). One bucco‐lingual section parallel to the long axis of the implant was obtained per specimen by a cutting and grinding technique (Donath & Breuner, 1982). With the sections reduced to a thickness of 120 μm, microradiographs (Figure 2a) were obtained (Faxitron X‐ray, Lincolnshire, IL, USA; 14 kV, 0.3 mA, 2.5 min; Insight Dental Film, Carestream Health Inc., Rochester, NY, USA) for measuring BMD in the surrounding of the implants (Huang et al., 2018; Karl et al., 2013). Following further reduction to a thickness of 70 μm and staining with toluidine blue O solution (Figure 2b) after preprocessing in 10% H_2_O_2_ solution, bone implant contact (BIC) was measured histomorphometrically (Huang et al., 2018, Karl et al., 2013) using a microscope (LEICA DM4B, LEICA Mikrosysteme Vertrieb GmbH, Wetzlar, Germany) equipped with a color image analyzing system (LEICA Application Suite, LEICA Phase Expert, LEICA Mikrosysteme Vertrieb GmbH).

### 2.4 Statistical analysis

In addition to compressive bone density testing in the cervical and apical part of an implant osteotomy (BP cortical and BP trabecular), the parameters determined were maximum implant insertion torque, primary implant stability, BMD, and BIC. Spearman rank correlation tests were used for describing potential correlations between different parameters. The level of significance was set at α = .05 for all statistical operations conducted.

## 3 RESULTS

All surgical interventions could be completed successfully, and healing following tooth extractions was uneventful. Due to insufficient vertical bone volume above the mandibular canal in two study sites, only a total of 14 implants instead of the envisaged 16 implants could be placed.

The mean values and standard deviations for all measurements conducted are given in Table 1. With the exception of implant stability measurements, the clinical measurements conducted showed high levels of variation resulting in considerable levels of standard deviation. The values for implant insertion torque and implant stability were not normally distributed, and consequently, Spearman rank correlation tests were applied.

**Table 1:** Results of clinical, microradiographic, and histomorphometric measurements performed

		Mean	*SD*
Clinical measurements	BoneProbe cortical	0.68	0.41
	BoneProbe trabecular	1.25	0.61
	BoneProbe overall	0.96	0.48
	Implant insertion torque (Ncm)	39.72	10.73
	Implant stability (ISQ)	69.04	6.24
Microradiograph	BMD (%)	75.36	5.59
Histomorphometry	BIC (%)	91.03	2.13

Abbreviations: BIC, bone to implant contact; BMD, bone mineral density.

In general, only weak correlations could be identified in the data set reaching significance only for two parameters, that is, BMD and BoneProbe (Tables 2a and 2b). A significantly positive correlation was found between the parameters BIC and BMD (Spearman's rho .53; *p =* .05) whereas a significantly negative correlation was observed between BMD and implant stability (Spearman's rho −.61; *p =* .03). Both BoneProbe measurements in the cortical and trabecular area positively correlated with implant insertion torque (Spearman's rho .60; *p =* .02). A slightly stronger correlation was observed between the average of both BoneProbe measurements and implant insertion torque (Spearman's rho.66; *p =* .01).

**Table 2a:** Results of Spearman rank correlation tests (Spearman's rho) for all parameters recorded in this study

	BIC	BMD	BoneProbe cortical	BoneProbe trabecular	BoneProbe overall	Implant insertion torque	Implant stability
BIC		.53	.23	.24	.17	.41	.15
BMD			−.09	−.05	−.04	.17	−.61
BoneProbe cortical				.68	.88	.60	.44
BoneProbe trabecular					.91	.60	.21
BoneProbe overall						.66	.29
Implant insertion torque							.31
Implant stability							

Abbreviations: BIC, bone to implant contact; BMD, bone mineral density.

**Table 2b:** Results of Spearman rank correlation tests (*p* values) for all parameters recorded in this study

	BIC	BMD	BoneProbe cortical	BoneProbe trabecular	BoneProbe overall	Implant insertion torque	Implant stability
BIC		**.05**	.44	.41	.56	.14	.63
BMD			.77	.88	.89	.55	**.03**
BoneProbe cortical				**.01**	**.00**	**.02**	.13
BoneProbe trabecular					**.00**	**.02**	.49
BoneProbe overall						**.01**	.33
Implant insertion torque							.30
Implant stability							

*Note.* Significant correlations are written in bold.

Abbreviations: BIC, bone to implant contact; BMD, bone mineral density.

## 4 DISCUSSION

This study resulted in the generation of baseline values for BoneProbe measurements in an intraoral animal model and tried to correlate clinical measurements of bone density and primary implant stability with the histologic parameters BMD and BIC for one specific bone level implant system. Due to a high level of variation observed in clinical measurements, the results presented should be interpreted with caution.

The only significant correlations among clinical measurements were found between compressive testing and implant insertion torque. This seems to be in line with previous studies in this field (Degidi et al., 2013; Huang et al., 2011; Wang, Lee, Wang, & Lin, 2015). Degidi et al. found partially contradicting correlations of insertion energy with histologic parameters of implant stability (Degidi et al., 2013) whereas Huang et al. found elastic modulus of trabecular bone and cortical thickness having an impact on primary stability but were not totally linearly correlated with insertion torque and stability measurements (Huang et al., 2011). Similarly, an increase in bone density or the presence of a cortical layer led to higher primary stability in an in vitro study, but the interrelationships among the measurements made remained unclear (Wang et al., 2015).

The benefit of intraoperative compressive testing of bone could be the possibility of adapting the surgical protocol (Sierra‐Rebolledo et al., 2016) in order not to overstress cortical bone (Duyck et al., 2015) trying to avoid bone damage leading to bone resorption (Eom et al., 2016; Menicucci et al., 2012). Another advantage of the BoneProbe besides being independent from a specific implant system might be that it allows for assessing cortical and trabecular bone separately, which takes into account that also trabecular bone may also contribute to primary implant stability (Dorogoy et al., 2017). However, threshold values for compressive tests defining different classes of bone are still missing.

Certain limitations have to be considered with respect to clinical transferability of this animal research. The intraoral minipig model allowed for using regular‐sized dental implants and represents a frequently applied test scenario (Catros et al., 2013) but limited vertical bone volume hindered from using all potential sites. In part, this was due to the bone reduction carried out after tooth extraction in order to achieve primary wound closure. Furthermore, only the status at implant insertion was evaluated, and consequently, no predictions can be made about osseous healing and potential resorption processes. As all clinical measurements were carried out by one single surgeon, repeatability of the measurements could not be checked as part of this experiment. Based on a previous study (Karl et al., 2019) using the BoneProbe in human cadaver bone, a reasonable level of repeatability and reliability could be assumed.

Within the limitations of this study and the weak correlations of BoneProbe measurements found with insertion torque values of a specific implant system, this diagnostic device may be useful for predicting implant stability at a stage where the clinician is still able to modify the surgical and the prosthetic treatment plan. Assuming that the weak correlations observed were due to the limited sample size, studies at a much greater scale involving various implant systems would however be required. Prior to clinical application, where the BoneProbe might assist in finding the optimal drill protocol, the optimal number of implants and the optimal loading scenario, a database would have to be created in order to define different bone classes.

## CONFLICT OF INTEREST

Matthias Karl discloses a conflict of interest as he holds a patent on the measurement principle of the bone density testing device used in this study.

## AUTHOR CONTRIBUTIONS

M. K. and T. G.‐K. conceived the ideas; V. P. and S. S. collected the data; all authors wrote and approved the manuscript.
